# Understanding public attitudes to death talk and advance care planning in Northern Ireland using health behaviour change theory: a qualitative study

**DOI:** 10.1186/s12889-022-13319-1

**Published:** 2022-05-06

**Authors:** L. Graham-Wisener, A. Nelson, A. Byrne, I. Islam, C. Harrison, J. Geddis, E. Berry

**Affiliations:** 1 Centre for Improving Health-Related Quality of Life, School of Psychology, Queen’s University Belfast, Belfast, UK; 2 Marie Curie Palliative Care Research Centre, Cardiff University, Cardiff, UK; 3 Marie Curie Northern Ireland, Belfast, UK

**Keywords:** Palliative care, Public health, Death and dying, Behaviour change, Advance care planning, ACP

## Abstract

**Objectives:**

Advance care planning is a key preparatory step in ensuring high-quality palliative and end of life care, and should be considered as a process, beginning with community-level conversations among lay persons. There is, however, indication that death talk among community-dwelling adults is not occurring, and there is a dearth of research examining why this is the case. This study aims to provide the first examination of barriers and facilitators to talking about death and dying among the general population in a UK region (Northern Ireland), and to provide a novel application of health behaviour change theory towards developing a theoretical understanding of the sources of this behaviour.

**Methods:**

The study involved qualitative analysis of responses (*n *= 381 participants) to two open-ended questions within a cross-sectional online survey, with recruitment via social media of adults currently living in Northern Ireland. Reflexive thematic analysis was conducted on open text responses per question, with the barriers and facilitators mapped on to health behaviour change models (the Behaviour Change Wheel COM-B and the Theoretical Domains Framework).

**Results:**

The findings evidence a myriad of barriers and facilitators to engaging in death talk, with themes aligning to areas such as lack of acceptance of death in social contexts and fear of upsetting self or others, and a need to improve interpersonal communication skills for facilitating conversations and improve knowledge of the existing services around death and dying. A theoretical understanding of the drivers of death talk is presented with findings mapped across most components of the COM-B Behaviour Change Model and the Theoretical Domains Framework.

**Conclusions:**

This study contributes to a small but emergent research area examining barriers and facilitators to talking about death and dying. Findings from this study can be used to inform new public health programmes towards empowering adults to have these conversations with others in their community towards upstreaming advance care planning.

## Background

In the UK, death rate and complexity of need for palliative and end-of-life care (PEOLC) patients are projected to increase substantially over the next two decades alongside the demand for PEOLC provision [1, 2]. This is reflective of international trends [3] representing increased provision of PEOLC as a global public health need. A key preparatory step in delivering high-quality PEOLC is advance care planning (ACP), cited across UK strategy documents and within quality indicators for good quality PEOLC [4–7]. ACP is an ongoing process that supports adults of any age in sharing their values, goals and preferences regarding future medical care during serious and chronic illness [8], and is evidenced to positively impact the quality of PEOLC [9]. There are however reports that indicate only a minority of adults in the UK have engaged in an ACP conversation [10, 11]. In acknowledging ACP as a continuous process across the life course [8] where an individual’s readiness for engagement may vary [12], there is value in considering how to “upstream” and “normalize” community-led conversations around death and dying more broadly [13].

The shift towards considering discussion of death and dying in the community aligns to the ‘new public health approach’ within palliative care [14]. The Health Promoting Palliative Care model (HPPC; [15]) advocates for movement towards a sustainable social model of end-of-life care, where death and dying are considered within the community context of everyday life and where each social actor is empowered to contribute. Advocates of HPPC recommend that at present building community capacity should be prioritized over further mainstream palliative care provision [16, 17]. A key principle of building community capacity includes normalizing death and preparing communities for end of life [17]. The mechanism for achieving this is through developing death literacy, defined as a set of knowledge and skills that make it possible to gain access to, understand and act upon end-of-life and death care options [18]. This includes the ability of individuals to provide talking support to a close friend, or child about death and dying [19], increasing readiness and providing a supportive context for community-based ACP conversations.

A significant proportion of UK adults report not being comfortable discussing death and dying with family and friends [20]. Most of the existing research on barriers to talking about death and dying has been conducted within the context of ACP with healthcare professionals (e.g. [21–23]), clinical populations of adults with a life-threatening illness (e.g. [24–26]), or with older adults (e.g. [27–29]). A recent call for future HPPC research resonates with the need to focus more “upstream”, stating the importance of approaching issues with the full population of interest, including ‘hidden publics’ and younger adults [14]. The authors recommend the use of surveys to evaluate the perceptions and experiences of the wider community, rather than only those defined as terminally ill [14]. Although there is an emergent evidence-base for the impact of new public health approaches to end-of-life care [14], a lack of observational research hampers capacity to address the dearth of high-quality interventions seeking early engagement with the general population.

In developing the evidence-base on which to inform interventions to increase talking about death and dying at a population level, it is important to develop a theoretically informed understanding of the target behaviour and associated change processes [30]. Authors have previously acknowledged the dearth of theoretically-informed interventions in PEOLC [31, 32]. Prominent within public health, health behaviour change theory including the Behaviour Change Wheel (BCW, [33]) and Theoretical Domains Framework (TDF, [34]) provide a systematic and theoretical basis for understanding and changing behaviour. In comparison to individual theoretical models, they enable a more comprehensive examination of a range of modifiable constructs, including internal factors and those pertaining to the external physical and social environment. The TDF and the inner part of the BCW, the Capability Opportunity Motivation-Behaviour model (COM-B), can be used to understand the Capability, Opportunity, and Motivational sources of any behaviour which can then inform the development of evidence and theory-based interventions. Behaviour change theory is increasing in use within PEOLC research (e.g. [35, 32, 36]) including application within a systematic review on implementation of ACP [37].

The question of how best to support listening to and incorporating individuals’ preferences around end-of-life aligns to unanswered research questions prioritised by people likely to be within the last few years of life, current and bereaved carers and healthcare professionals [38, 39]. With exception (e.g., [40]), there is limited research examining barriers and facilitators to talking about death and dying in general population samples in the UK. There is however recognition of the importance of building community capacity in providing PEOLC, including from the perspective of specialist palliative care providers [41] and general practitioners [42]. This study aims to provide the first exanimation of barriers and facilitators to talking about death and dying in a UK region (Northern Ireland). Secondly, the study aims to provide a novel application of health behaviour change theory towards informing future evidence-based interventions to increase discussion of this important topic in the general population.

## Methods

### Design and setting

The study involved qualitative analysis of responses (*n* = 381 participants) to two open-ended questions within a cross-sectional mixed-methods online survey. Although underutilized, mixed-methods/qualitative surveys have been recommended as a ‘best fit’ when seeking multiple perspectives from large populations, when the topic suits a ‘wide angle lens’, and when wishing to encourage disclosure and participation in regards to a sensitive topic [43]. Reporting of findings is informed by CHERRIES guidance for reporting Internet surveys [44] and COREQ guidance for reporting qualitative research [45].

The setting for the study is a region of the United Kingdom, Northern Ireland (population circa 1.9 million). In Northern Ireland, the local death rate is projected to increase by 31 per cent by 2031 [1], as calculated pre-pandemic, the highest proportional increase across the UK nation states. A recent survey conducted in 2019 indicates only a minority of the population (7%) have previously engaged in an ACP conversation [11].

### Sampling and recruitment

The sampling frame for this study defined community as a member of the public currently living in Northern Ireland. Eligibility criteria included adults (≥ 18 years of age) who have the capacity to express their opinion.

Convenience sampling was used with an open-survey link shared via social media (Twitter & Facebook) by the research team using a dedicated handle (@PADDNI_Research). Several organisations (e.g., charities, public-facing bodies, private businesses) were invited to share the survey link and were provided with posters to display on their premises. Participants were provided with a participant information sheet informing them of the purpose of the study, approximate survey completion time, which data are stored, where, and for how long, and details of the research team. Participants completed an informed consent statement prior to completion of the survey and provided informed consent for use of their data again at survey completion [46]. Participants were not contacted individually. No personal data were collected. Furthermore, participants had no prior relationship with the researchers. This study was approved by the QUB EPS Faculty Research Ethics Committee.

### Survey Design and Implementation

The survey was originally developed by the co-authors at Cardiff University [47] in collaboration with the One Wales Palliative Care Program of the National End of Life Care Board, informed by findings from the James Lind Alliance Palliative and End of Life Care Priority Setting Partnership [38, 39]. The aim of the survey was to understand attitudes towards death and dying, with included domains on fears about death and dying, preferences and priorities around EoLC, knowledge around terminologies commonly used in EoLC and understanding about ACP, EoLC plans and communication around death and dying. An extensive literature review informed the domains of interest and the survey questions were refined by a group of experts in this field, including volunteer research partners. Minor modifications were made by the research team to the original survey for the Northern Ireland context, with input of clinician and policy colleagues at Marie Curie Northern Ireland.

The 41-item online survey was hosted on Qualtrics [Qualtrics, Provo, UT], with items presented to participants across 7 pages (range 1–17 items per page) and in a standardized order. Respondents were able to review and change their answers, and in line with guidelines [46] a withdraw button was included on each page. There were no forced responses to items. No incentives were offered to participants. Prior to data collection, the survey was piloted with 12 participants with no amendments made. Data was collected between 20^th^ January and 18^th^ April 2019, and the median time of survey completion was 14.2 min.

Towards understanding communication around death and dying, this study is a focused qualitative analysis of two open-ended survey items from the larger survey-

i) “*As a society, how much do we talk about death and dying in Northern Ireland? If not enough, how do you think this can be increased*?” (Question 5)
ii) “*Is there anything that prevents you from talking about death and dying? If yes, please state”* (Question 14)

### Analysis

Data were exported from Qualtrics [Qualtrics, Provo, UT] to a Microsoft Word Document. In total, 924 respondents consented to participate in the survey, after 2 respondents were removed as providing multiple responses (identified by Qualtrics as multiple identical responses from same IP address < 12 h). Data were scrutinized for i) duplicate responses by examining sociodemographic variables (age, gender, education) alongside responses, and ii) responses completed in < 5 min with participants withdrawing at first opportunity, with 2 responses removed. In total, 61% of responses (*n* = 562) completed the survey by providing informed consent for their data to be used at the end of the survey. A total of 381 participants provided responses to the two open-ended questions analysed in this study: with 179 responses to the barriers question and 326 responses to the facilitators question.

This study adopts a subtle realist epistemological stance within an interpretative paradigm, which recognises the subjectivity of human experience but is concerned with identifying common patterns across subjective narratives which denote collective experience. Reflexive thematic analysis was conducted on open text responses for each question separately using Microsoft Word and Microsoft Excel to track codes and notes [48]. This method of analysis fitted the subtle realist stance and provided sufficient flexibility to explore the range of perspectives and experiences conveyed by a large cohort of participants to address the broad research aim [43]. This followed a six-stage process which includes familiarisation with the data; inductive coding; exploring potential themes; reviewing and confirming themes; labelling and defining themes; and reporting and interpretation of themes [48]. Codes were identified by a lead analyst (JG, BSc) and were iteratively reviewed to ensure that these accurately captured relevant units of data [49]. A coding tree was developed from confirmed codes in table format on MS Word, which guided the identification of subthemes and overarching themes. Themes/subthemes were iteratively constructed and discussed with the wider analysis team (LGW, PhD & EB, PhD) and were cross-checked with codes and quotes to ensure that these remained closely bound to the data. Final themes/subthemes were established following rigorous discussion. In line with recent recommendations, the reflexive thematic analysis was conducted inductively, with no theoretical constraints on the identification of themes from the COM-B and TDF [50]. Team meetings were held frequently throughout the analysis to reflect on progressive interpretation of the data and reflexive notes. This ensured that reflexive practice was maintained, supporting a transparent and credible analytic process [49]. Barriers and facilitators were then mapped onto the COM-B [33] and the TDF, where they were deemed to conceptually fit [43]. This mapping process involved developing descriptions derived from themes/subthemes, which are aligned with the corresponding model components. This process also facilitated translation of themes/subthemes into practical language for aid of interpretation. Researchers JG, LGW, and EB were involved in this conceptual mapping process. JG is a psychology student with an interest in research, health psychology, and wellness-promoting behaviour change, however, has no personal or professional background with palliative care. LGW has a professional background in health behaviour change, and personal and research experience in palliative care. EB has a professional background in health behaviour change, however has no prior personal or professional affiliation with palliative care.

## Results

Participant demographics are detailed in Table 1. The majority of respondents were aged 25–64 years of age, were female, of a white ethnic group, and were living with a partner or spouse. A small proportion were living with a chronic physical condition (18%), a mental health condition (9%), and/or a disability (14%). Over half declared that they identified as non-religious, although almost all reported that they were raised under a certain religion. Most participants were educated to at least graduate level and a small number had migrated to Northern Ireland (the majority of which had resided in NI for more than 15 years).

**Table 1:** Participant socio-demographic characteristics (*N* = 381)

**Age**	**Freq (%)**
18–24	30 (7.9%)
25–34	56 (14.7%)
35–44	76 (19.9%)
45–54	86 (22.6%)
55–64	91 (23.9%)
65–69	17 (4.5%)
70–74	16 (4.2%)
75–79	6 (1.6%)
80–84	2 (0.5%)
85 +	1 (0.3%)
**Gender**	
Male	90 (23.6%)
Female	290 (76.1%)
Other	1 (0.3%)
**Ethnicity**	
White	372 (97.6%)
Pakistani	1 (0.3%)
Mixed ethnicity	5 (1.3%)
Other ethnicity	3 (0.8%)
**Relationship status**	
Single	75 (19.8%)
Married/partner	248 (65.4%)
Divorced	31 (8.2%)
Separated	11 (2.9%)
Widowed	14 (3.7%)
**Chronic physical health condition**	
** Yes**	68 (18.2%)
**Chronic mental health**	
Yes	31 (9%)
**Disability**	
Yes	52 (13.8%)
**Religion (current)**	
None	205 (53.9%)
Christian (of no/different denomination(s))	100 (26.3%)
Roman Catholic	132 (34.9%)
Non-Christian	7 (1.9%)
**Religion (brought up with)**	
None	44 (11.6%)
Christian (of no/different denomination(s))	195 (51.5%)
Roman Catholic	132 (34.9%)
Non-Christian	7 (1.9%)
**Education**	
Primary	1 (0.3%)
Secondary	70 (18.4%
Graduate	310 (81.3%)
** Emigrated to Northern Ireland from another country**	50 (14.9%)
(*of which*) duration of time living in Northern Ireland > 15 years	35 (10.4%)

## Barriers to communication about death and dying

Three overlapping themes were constructed for the question surrounding barriers: ‘Apprehension at navigating conversations’; ‘Emotional responses to death talk’; and ‘Unacceptance of death talk in different social contexts’. See Table 2.

**Table 2:** Barriers coding tree of themes and illustrative quotations

Theme	Subtheme	Illustrative Quotation
**Apprehension at navigating conversations**	Challenge of sensitively navigating conversations about death	*I don’t want to sound insensitive* (Participant 249, F, 25–34 years)
	Concern over ability of others to facilitate conversations about death	*Bringing up the topic either makes others uncomfortable or dismiss it with a short "Sorry for your loss*” (Participant 847, F, 18–24 years)
**Emotional responses to death talk**	Conversations hindered by own emotions	*I get upset about it—don't want to make people uncomfortable* (Participant 862, F, 18–24 years)
	Perceived risk of arousing challenging emotions in others	*Fear of making others uncomfortable or upset (Participant 18, F, 35–44 years)*
**Unacceptance of death talk in different social contexts**	Societal norms sustain lack of integration of death talk	*It's just not done here, I am not sure why* (Participant 333, F, 35–44 years)
	Cultural beliefs can deter openness about death	*The discomfort of others regarding the topic. I am viewed as very strange for wanting to discuss “such negative topic”, but it’s important to me. I have a different philosophy and spirituality than those I love, which they struggle with* (Participant 398, F, 55–64 years)
	Perception that known others are unwilling to engage in death talk	*My family don’t want to talk about it* (Participant 259, F, 70–74 years)
	Perception that death should only be discussed with family and close persons	*I don’t like to share my personal feelings with people I am not close to* (Participant 494, F, 35–44 years)

### Apprehension at navigating conversations

This theme relates to the challenge of talking about death with others. There was an assumption that death talk needs to occur in a supportive context and thus requires skilled communication. Participants conveyed concerns about their own ability or the ability of others to orchestrate conversations about death in a sensitive manner. This theme comprises two sub-themes: 1) Challenge of sensitively navigating conversations about death 2) Concern over ability of others to facilitate conversations about death.

Subtheme one captures the perspectives of participants who feel they lack the skills and confidence to instigate and sustain conversations about death when approached by others affected by death. These participants felt concerned about offending others, saying something inappropriate, or prompting discussion at an inappropriate time or in the wrong context. There was general apprehension about *how* death talk should take place.

*Saying the 'wrong thing'* (Participant 483, M, 65–69 years)

*If someone has recently experienced loss I would be cautious to raise the subject* (Participant 739, F, 18–24 years).

*If the person is older and I don't know how comfortable, able, they are to discuss death* (Participant 90, F, 45–54 years).

Subtheme two describes the perception that other people are not always helpful in supporting conversations about death. This refers to the perceived ability of other people to instigate or host constructive discussions about issues/events related to death. Participants’ often reflected that this can result in reluctance to open up to or confide in others. This is despite underlying wishes that more helpful conversations could take place at times of need.

*People seem at a loss for words and I feel quick to soothe them by brushing it off or minimising it. I suppose there is an awkwardness about death.* (Participant 101, F, 18–24 years).

*If I’m caring for someone that is end of life and wishes not to talk about it. Or I perceive it to be inappropriate, I let the other person lead the conversation. Sometimes in a social context of I talk about personal bereavement I don’t want to be met with pity, sometimes it’s just a factual thing or sometimes it’s not information I wish to share* (Participant 32, F, 25–34 years).

### Emotional responses to death talk

This theme captures how personal emotions, or the perceived emotional response of others can hinder honest conversations about death. In particular, ‘fear’ was commonly referred to by many participants, and emotions including fear and upset are observed across each of the subthemes constructed: 1) Conversations hindered by own emotions 2) Perceived risk of arousing challenging emotions in others.

In the first subtheme, participants reflected on how their own emotional reactions to death prevented them from discussing death frankly with others. Participants worried that expressing feelings of sadness and emotional distress could result in the discomfort of others or might be perceived as inappropriate in certain contexts. Many participants also described how it is often difficult to talk to others when they are personally affected by death because expressing feelings is psychologically stressful. Participants also discussed how it ‘scares’ them to ‘think about’ death.

*Knowing that I will cry and sometimes that's not acceptable or useful in certain scenarios* (Participant 820, F, 45–54 years).

*It’s difficult to talk about your own experiences. I had a very traumatic death of a sibling and received little support, although I’m now able to talk about it this has taken ten years to be able to (Participant 468, M, 25–34).*

*It scares me to think about it* (Participant 507, F, 35–44 years).

Subtheme two describes participants’ perceptions that talking about death can trigger challenging emotions in others, so there is an implicit assumption that death talk is more harmful than helpful. This deters individuals from initiating conversations about death. Participants felt that emotional reactions to death may differ across individuals and social contexts, which makes it difficult to recognise when these discussions are appropriate and acceptable (this links with theme three).

*At times you want to avoid upsetting someone even though you know it would be good for them to talk* (Participant 39, F, 45–54 years).

*Many close family and friends fear death, and don’t like…to talk about it openly* (Participant 54, F, 55–64 years).

*Getting tearful, upset, upsetting others. Making things worse.* (Participant 415, F, 45–54 years).

### Unacceptance of death talk in different social contexts

This theme captures how conversations about death are uncommon at a societal level, which sustains lack of acceptance within communities and social circles. The rationale for lack of acceptance relates to normative behavior, cultural diversity, and assumptions about the appropriateness of death talk. Four subthemes were identified for this theme: 1) Societal norms sustain lack of integration of death talk 2) Cultural beliefs can deter openness about death 3) Perception that known others are unwilling to engage in death talk 4) Perception that death should only be discussed with close others.

Subtheme one describes how conversations about death are not commonplace and scarcely feature in social interactions, except for in certain times and contexts, such as when a person is at the end of their life or in the case of grief. Even then, death talk is not widely or openly practiced. Death is perceived to be a negative topic and this belief is perceived to be embedded in our “*culture*” at a societal level.

It’s just not done here, I am not sure why (Participant 333, F, 35–44)

*I just don’t think it’s socially acceptable—people think you are in a bad mood or always thinking the worse if you talk about negative things such as dying* (Participant 832, F, 18–24 years).

Subtheme two relates to participants’ concerns about death talk causing offence or distress to people who have strong spiritual or “religious beliefs”. Religious or spiritual beliefs/values may hinder social interactions about death because of perceived incongruity of perspectives. This subtheme interconnects with theme one as this relates to perceived ability to discuss death in a sensitive and respectful manner.

*My family being religious while I am not and I would want different things when I die than they would expect* (Participant 859, F, 25–34 years).

*If it outwardly upsets the other person or if I don’t know people very well. I am very aware that a lot of people have a very closed attitude to end of life due to their religious beliefs* (Participant 238, F, 35–44 years).

*Fear that I am making them talk about issues they are not comfortable with even though I am. Fear of insulting someone's core beliefs* (Participant 328, F, 35–44 years).

The third subtheme relates to participants’ beliefs that known others such as family, friends, and colleagues are often not willing to discuss death. Participants felt that death talk is not typically welcomed by others in their social circle. Rather, other people tend to avoid the subject and there was a sense that one might be regarded as “strange” for “wanting to discuss” it. It is this lack of inclination to facilitate discussions that may prevent people from opening up about death when they need to.

Other people’s negative attitudes, I get shut down by some family members who find it hard to talk about (Participant 289, F, 18–24)

*When the other person doesn’t want to. My mum didn’t want to know she was dying and wouldn’t listen to any prognosis or treatment options* (Participant 816, F, 35–44 years).

Subtheme four captures participants’ perspectives that death talk is only acceptable when with close others such as family or professionals whom people feel close to. Participants felt that the sensitivity of the subject can restrict who they talk to about death and the contexts in which these conversations can happen. This interconnects with theme one as participants imply that trust and rapport are important prerequisites for discussions about death.

*This is a personal topic which l would discuss only in particular circumstances and with particular people. Should l be in a position where health professionals are involved, l would be comfortable discussing death/dying only with a person with whom l felt at ease* (Participant 732, F, 70–74 years).

*Would need to be with someone close. Hard topic to discuss with an acquaintance or someone I am not close with* (Participant 395, F, 35–44 years).

### Facilitators to enhance communication about death and dying

Four overlapping themes were constructed for the question surrounding facilitators: ‘Increasing knowledge of the ‘death system’; ‘Improving interpersonal communication”: Encouraging acceptance of the need for death talk’; ‘and ‘Groups and Individuals with ability to promote the discussion’. See Table 3.

**Table 3:** Facilitators coding tree of themes and illustrative quotations

Theme	Subtheme	Illustrative Quotation
**Increasing knowledge of the ‘death system’**	Improving information provision	*Provide more information to patients and families on their rights and choices to help guide conversations so that people can decide and opt for what is best for them/what they want (Participant 36, F, 18–24 years)*
	Education along the life course	*Firstly being taught in schools. We learn about birth but not about death…. it’s still treated like a taboo subject and as a result nobody is prepared for it.. (Participant 305, F, 35–44 years)*
	Experts sharing their experience	*Using similar campaigns which raised awareness of other social issues in the past. Also finding people who are willing to share stories and the facts….carers and the professionals (Participant 82, F, 55–64 years)*
**Improving interpersonal communication**	Accessible communication from healthcare providers	*Health professionals be more direct when talking about death to paitents and families (Participant 324, F, 45–54 years)*
	Practical support to improve interpersonal communication skills	*Not sure, people don’t know what to say. Too much emphasis on being positive when terminally ill (Participant 119, F, 45–54 years)*
	Increasing awareness of different belief systems	*Make it less medical so target the whole population on neutral footing. Ie not based on religion or beliefs but person centred and individual (Participant 32, F, 25–34 years)*
	Acknowledging individual responsibility in initiating discussions	*By each person talking to families and friends about their own feelings/wishes about dying AND (harder to do I think) asking others what their views/feelings/wishes are regarding their demise- not in general—specifically about their own case (Participant 474, F, 55–64 years)*
**Encouraging acceptance of the need for death talk**	Raising awareness of relevance across people and contexts	*Change attitudes by advertising how easy it can be and the benefit it is when we all know what is to happen at the end of life (Participant 71, F, 55–64 years)*
	Addressing fear surrounding discussion of death and dying	*By encouraging people to talk about their experience, take away the superstition that it's bad luck to talk about death! (Participant 158, F, 35–44 years)*
	Normalising death as a part of life	*If the topic is introduced in schools, with death being treated as a natural part of our lifecycle, a lot of the barriers and fears can be overcome (Participant 42, F, 55–64 years)*
**Groups and Individuals with ability to promote the discussion**		*More awareness, news programmes, newspaper articles, social media *etc. *(Participant 6, F, 65–69 years)*

#### Increasing knowledge of the ‘death system’

This was a prominent theme which relates to how enhancing knowledge and increasing opportunities to build understanding about key terminology and processes surrounding dying and death would be a useful starting point to augment communication about the topic in different contexts. Three subthemes were constructed: 1) Information provision, 2) Education along the life course and 3) Experts sharing their experience.

The first subtheme information provision refers to the need to increase the quality and availability of informational resources to equip people with the basic knowledge to understand their options at the end of life and key terms/processes relating to areas such as palliative care and end of life care. This relates to tangible resources such as pamphlets and adverts tailored to different contexts and across different demographic groups.

*Provide more information to patients and families on their rights and choices to help guide conversations so that people can decide and opt for what is best for them/what they want (Participant 36, F, 18–24 years).*

*I think that although in different social and different backgrounds, the topic of death is viewed differently, hence making a generalised fact sheet or ‘black and white’ explanation void. (Participant 79, F, 25–34).*

In subtheme two, participants described the ‘*taboo*’ around discussion of death and dying as a social construct which develops across the life course, with opportunity to normalize discussion through ‘*early*’ intervention. Participants stressed the importance of embedding discussion with children and adolescents into formalized curricula within primary and secondary education, towards achieving parity with ‘*career advice or sexual care*’. Several developmentally appropriate topics were suggested, with need for discussion on grief and loss identified for younger children.

*I feel death should be talked about more openly with children from a young age and it should not be a taboo subject that we hide from them. This should happen in the home and in schools. This will help prepare them should they face bereavement as a child or an adult. Having this knowledge may reduce the unavoidable shock and grief that individuals have to deal with at some stage of the life-course (Participant 214, F, 45–54 years).*

*Start earlier, include health and well-being on a school curriculum that includes issues related to death and dying—and life. Organ donation is important in its own right and also is a doorway into conversations about mortality (Participant 80, M, 55–64).*

Although the contribution of formalized education was discussed in relation to schools and universities, the role of ‘education’ as empowering rather than simply information-provision (as in subtheme one) was frequently described in reference to adults. A key emphasis was placed on emotional preparation across the life course, in terms of bereavement at all stages and with advance care planning cited for older adults. The need for more “*public discussion”* was described, taking the form of workshops or seminars. Participants emphasized the need for there to be ownership from organisations perceived to be experts in this area (medical charities provided as examples), and the opportunity to harness existing communities such as workplaces to embed discussion.

To remove the fear and negativity around death and dying, the subject needs to be living outside where the public live (Participant 716, M, 70–74 years)

In subtheme three there is the perception of ‘privileged knowledge’ existing in relation to death and dying, and the need for ‘experts’ across various sectors to act as knowledge brokers towards sharing process and experience-based knowledge with the aim of addressing the “*mysteries*”, “*myths*” and “*uncertainties*”. The importance of fostering realistic expectations about end of life care was emphasized.. A diverse range of expertise was recognized, including those working in healthcare, funeral services, finance, and individuals with lived experience of the death system.

More public discussion. Encouraging health care professionals in particular to speak without fear about the processes they participate in (Participant 385, M, 65–69 years)

*Using similar campaigns which raised awareness of other social issues in the past. Also finding people who are willing to share stories and the facts….carers and the professionals. (Participant 82, F, 55–64).*

### Improving interpersonal communication

This theme raises the importance of compassionate and person-centered communication about the topic of death and dying and provides a sense of what aspects of communication are especially important, why these are important, and who should instigate conversations about death and dying. Four subthemes were identified: 1) Accessible communication from healthcare providers, 2) Practical support to improve communication skills, 3) Increased awareness of individual differences, 4) Acknowledging individual responsibility in initiating early discussions.

Subtheme one describes the need for “*open and caring*” communication from healthcare professionals in respect to advance care planning. There was a general perception that a culture change is needed regarding communication about death and dying in health contexts, whereby optimising access to specialist palliative care and embedding palliative care approaches within generalist health and social care structures will facilitate “*earlier*” and more “*routine*” conversations. The importance of training and support for healthcare professionals was also described. The importance of getting to know the patient through provision of anticipatory care to ensure future care is person-centred was emphasised.

*In most circumstances death & dying are anticipated, usually in a healthcare setting. This presents an opportunity for healthcare professionals to broach the subject with their patients and families. I feel that this should become a more 'routine' conversation in clinical care. It is too often discussed at a very late stage which may only add to a sense of stress and reduce the time available for careful reflection. This would require something of a culture change in healthcare, adequate training in communication could help this. (Participant 817, M, 25–34)*

By health professionals setting the bar and talking about it in an open and caring way (Participant 18, F, 35–44 years)

Subtheme two describes the belief that increased interpersonal support and communication skills training for people across demographic groups could increase an individual’s capacity to facilitate death talk. This relates to supporting individuals to have more ‘open and honest’ discussions, including the need to avoid the use of euphemisms around death or being overly positive. Providing advice on “*conversation openers*” was recommended, with organ donation cited as a useful anchor to initiate conversations, including discussion into different types of deaths. Participants emphasized a need for skill development around how to compassionately listen when discussion is instigated by others on death and loss, with recognition this is a “*learned skill*”. A particular focus was placed on developing an awareness of helpful and unhelpful responses, with the latter serving to limit in depth discussion.

*A public health campaign promoting awareness of why we need to openly talk about death. Workshops e.g., for parents to learn how to talk to children about death. Classes in schools that contain an element of discussion about death (Participant 367, F, 45–54 years).*

Teach younger people how to discuss sympathetically as this is learned skill (Participant 406, M, 55–64)

Subtheme three suggests that to facilitate a supportive conversation about death and dying it may be helpful to avoid dogmatic topics which may arouse conflict and discomfort. Rather, there is emphasis on the need to talk about death and dying from the perspective of “*human understanding*”, to enable participation of people from diverse belief systems. Emphasis was placed on religious organisations as having ownership of discussion around death and dying, with a focus on the afterlife rather than end of life.

*By normalising it. Making it ok for dying people to talk about death. Children be informed maybe in a non-religious way. Acceptance of the pain of death alongside the inevitability. Maybe it'd help people be more grateful of life (Participant 496, F, 45–54 years).*

By encouraging honest discussion without moral or religious judgement (Participant 785, F, 45–54 years)

Subtheme four relates to the need for individuals to acknowledge that they have a personal role in initiating or engaging in conversations about death and dying, however challenging. Such conversations were perceived to begin at the familial level, with a need for open discussion around wishes and feelings towards death and dying. The importance of not excluding younger family members from these discussions was emphasised, with a need for “*healthy conversations from childhood throughout adulthood*”.

*By each person talking to families and friends about their own feelings/wishes about dying AND (harder to do I think) asking others what their views/feelings/wishes are regarding their demise- not in general—specifically about their own case (Participant 474, F, 55–64 years)*

I think we need to be brave and start the conversations with our family, especially in aspects of organ donation wishes and end of life care (Participant 10, F, 35–44 years)

Participants described how developing plans for end of life should be embedded within these supportive conversations, which need to happen early, prior to rapid decline of health in oneself or others. In addition to establishing preferences around end of life care, the opportunity for individuals to plan their own funerals and develop living wills was also articulated.

We encourage pregnant women to have birth plans so why can’t we normalise the death plan (Participant 816, F, 35–44 years)

*Encouraging acceptance of the need for death talk* This theme explores the need to increase the acceptability of engaging in supportive conversations about death and dying, as a precursor to the other themes. Three subthemes are contained within this: 1) Raising awareness of relevance to different populations 2) Addressing fear surrounding discussion of death and dying 3) Normalise death as a natural part of life.

The first subtheme describes how it is necessary to help individuals to understand why talking about death and dying might be relevant for them, across different stages of life and diverse contexts. Recognising the relevance may promote a more open attitude towards engaging in such conversations. The need for health promotion campaigns was emphasized. Alongside this was the perception that certain deaths are seen as more socially acceptable, with other deaths more likely to not be discussed. It was described how parity is needed in changing attitudes towards the discussion of deaths relating to child/baby death, suicide, drug addiction and clinician-assisted death.

*I feel we only talk about death after the event and not in forward planning. We need people to accept and understand that critical illness can affect us at any age (Participant 238, F, 35–44 years).*

Healthcare conversations about end of life should be introduced in middle age (Participant 712, F, 25–34)

Subtheme two focuses on the need to support individuals who feel afraid to initiate or engage in discussions around death and dying. Participants described a perceived relationship between “*taking about dying and hastening death*”, and an associated belief that “*if we don’t talk about it, it is not going to happen*”. The need to help individuals disassociate superstitious fears was emphasized. Fear is recognized as a prominent emotional barrier and thus strategies to reduce fear should be explored.

Not sure. It's a societal thing, people fear that by talking about it, it will somehow bring death to them (Participant 134, F, 35–44 years)

By encouraging people to talk about their experience, take away the superstition that it's bad luck to talk about death! (Participant 158, F, 35–44)

Subtheme three explores the usefulness of promoting acceptance of death and dying as a natural part of the lifecycle, not to be stigmatised but rather understood. This includes changing attitudes away from “*the belief that talking about death is morbid*” and rather encouraging society to be “*death positive*”. Participants referred to death cafes as being a useful facilitator of a positive attitude to death, along with using death to facilitate a focus living meaningfully.

*I think when you compare the polarity between birth and death of how much it is acknowledged its bizarre. Death is just as big a part of life. Yet there is zero sense of belonging or community in it. It feels like a stigma. Taboo. Keep hush. It should be as easily spoken about as a birth or even like a wedding. It’s such a knee jerk reaction to cower away from it when if we could all embrace it and bring a sense of community camaraderie to it, it wouldn’t be as dark and frightening (Participant 316, F, 25–34 years).*

Prevalent within this subtheme was the need to change protectionist attitudes towards discussing death and dying with children and younger family members, “*it should not be a taboo subject we hide from them*”. Emphasis was placed on engaging openly and honestly with children around death, and not dismissing or explaining death is a reductionist manner. Participants also described the need for society to encourage inclusion of children within death rituals such as funerals and wakes, and to not hide serious illness or grief to normalise emotional reaction to death.

*By not excluding children from discussing the death/ funerals of elderly relatives and pets, often a way to show that death is a part of living and importantly allowing them to express their emotions (Participant 270, F, 70–74 years).*

### Groups and Individuals with ability to promote the discussion

This overarching theme captures the variety of stakeholders, services, and approaches with potential to facilitate greater communication about death and dying. Sources yielded through a content-based extraction of key communicative tools/sources include: 1) Media 2) The Arts 3) Experts 4) Service Users 5) Third Sector 6) Healthcare providers 7) Individuals 8) Schools 9) Policy makers 10) Researchers. The capacity to endorse greater communication about the topic broadly relates to utilizing a range of vessels/resources to (considering the issues raised in previous themes) educate individuals e.g. schools or charities providing workshops/talks, raise awareness e.g. emotive social media posts/images, normalize e.g. open discussions instigated within families, addressing challenging emotions like fear through theatre/film or service users sharing their experiences. Within this theme, the role of policy makers is also discussed, suggesting that there is potential to augment communication about death and dying across various levels of society.

### Integrated findings

**Fig. 1:**
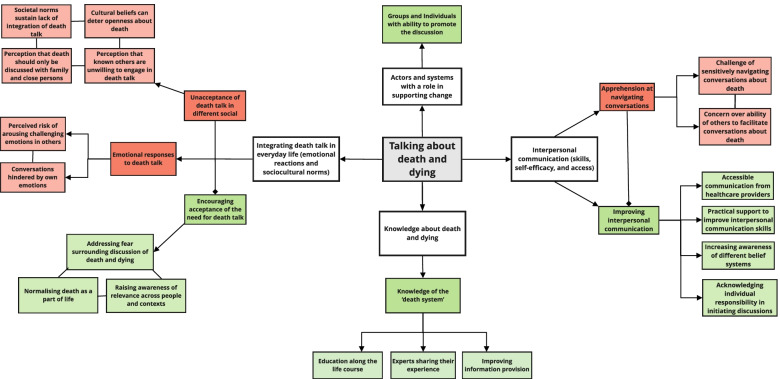
Cross-cutting constructs identified across barrier and facilitator themes

As illustrated by Fig. 1, the barriers and facilitators when integrated can be conceptualised to relate broadly to interpersonal communication, actors and systems with a role in supporting change, knowledge about death and dying, and integrating death talk in everyday life. This illustrates core constructs which interventions can be designed to target, by framing the perceived barriers to talking about death and dying in the context of prospective solutions to mitigating these.

### Barriers and facilitators mapped to Behaviour Change Theory

The barriers and facilitators to talking about death and dying mapped to most constructs within the COM-B and TDF. Table 4 presents the barriers and facilitators translated into a series of descriptions which are aligned with conceptually relevant COM-B and TDF constructs.

**Table 4:** Barriers and facilitators mapped to the COM-B and TDF

COM-B Component	TDF Domain	Barriers	Facilitators
Psychological Capability	Knowledge	Lack of understanding of how to engage in death talk in a way that is helpful and supportive (*overlaps with apprehension at navigating conversations*) Uncertainty about when and with whom it is appropriate to engage in death talk (*overlaps with social role/responsibility*)	Increase quality and availability of tangible information resources (tailored to different contexts & groups) to facilitate understanding of options at the end of life and key terms/processes Experts across various sectors to share process and experience-based knowledge of the death system (*overlaps with social influences*) Provide education along the life course to empower and emotionally prepare individuals
	Skills	Lacking the interpersonal skills to facilitate constructive (i.e. sensitive and culturally respectful) conversations about death (*overlaps with beliefs about capabilities*)	Increase interpersonal support and communication skills training for people across demographic groups to facilitate death talk Support individuals to talk about death and dying in a way which enables participation of people from diverse belief systems
	Memory, Attention and Decision Processes	-	-
	Behavioural Regulation	-	-
Physical Capability	Skills	-	-
Social Opportunity	Social Influences	Perception that other people are unwilling to engage in constructive conversations around death Death is not commonly discussed within NI culture and religious diversity invites sensitivity toward death talk	Normalize death talk through early intervention by embedding discussion with children and adolescents within formal education and the family (*overlaps with intentions*) Harness existing communities such as workplaces to embed discussion such as death cafes
Physical Opportunity	Environmental Context and Resources	Conversations about death with close others occurring at a late stage and this timing issue is a challenge	Optimise access to specialist palliative care and embed palliative care approaches within generalist health and social care structures to facilitate earlier and more routine conversations
Reflective Motivation	Social/Professional Role and Identity	Death talk is perceived to be only appropriate within the context of families or with professionals	Encourage individuals to acknowledge they have a personal role in initiating and engaging in conversations around death and dying (*overlaps with intentions*) Provide training and support for healthcare professionals to have more routine ACP conversations (*overlaps with skills*)
	Beliefs about Capabilities	Individuals believe they are not capable of discussing death in a helpful way (overlaps with skills	Provide guidance on ‘conversation openers’ to facilitate individuals to instigate conversations
	Beliefs about Consequences	Individuals believe that discussing death will result in social and/or personal discomfort (*psychosocial repercussions*)	Help individuals to understand why talking about death and dying might be relevant to them, at different stages in life Help individuals to understand the benefits of developing plans for end of life at an early stage, within supportive conversations Help individuals to develop a positive attitude to death, by using death to facilitate a focus on living meaningfully
	Intentions	Assumption that others generally are not willing to/ do not want to discuss death	Help individuals to recognize that others wish to have conversations around death and dying
	Goals	-	-
Automatic Motivation	Optimism	-	-
	Reinforcement	-	-
	Emotion	Anticipated emotional reactions hinder conversation about death *(unpleasant emotions underpin social and personal barriers)*	Help individuals to disassociate superstitions and to address fear around talking about death and dying

Relevant constructs for both barriers and facilitators include ‘Psychological Capability’ under the TDF domains ‘knowledge’ and ‘skills’, where facilitators described strategies to address the knowledge and skills barriers identified. ‘Social Opportunity’, within the TDF domain ‘social influences’, was also relevant, where facilitators described strategies to adjust existing social norms (i.e. increasing opportunities to normalize discussion). Another pertinent construct was ‘Reflective Motivation’, within the TDF domains ‘social/professional role/identity’ and ‘beliefs about consequences’. Barriers described apprehension about whether social role/identity restricted death talk and causing discomfort as result of death talk. Whereas, facilitators described ways to enhance professional responsibility in health contexts and increasing perception of the benefits and relevance of discussions about death across the lifecycle. Related to this, the construct ‘Physical Opportunity’ was mapped to facilitators within the TDF domain ‘Environmental Context and Resources’, and described the infrastructural change needed in health systems to increase opportunities for death talk. Lastly, ‘Emotions’, under the construct ‘Automatic Motivation’ was also relevant, where facilitators described ways to address challenging emotions and the perception of arousing challenging emotions in others.

Less relevant constructs appeared to be ‘Physical Capability’ (COM-B), several of the TDF domains under ‘Psychological Capability’ (Memory, Attention and Decision Processes & Behavioural Regulation), one domain under ‘Reflective Motivation’ (Goals), and two domains under ‘Automatic motivation’ (Optimism and Reinforcement).

## Discussion

This study contributes to an emergent evidence towards understanding the barriers and facilitators to talking about death and dying for the general population. Previous research identifies several barriers including lack of knowledge of the death system [51], fear/distress associated with thinking about death and dying [52] and difficulty engaging others in death talk or fear of upsetting others [53]. An aligned Welsh study [47] conducted at a similar period of time to the current study identified several levels of barriers, including social perception and practice (e.g. death as a societal ‘taboo’), lack of opportunities (e.g. perception of no family & friends to talk about this with) and support and personal emotions and values (e.g. concern over causing distress). The Welsh study also identified several facilitators, such as enhancing acceptance of death as a part of life and using a public health approach to engage the public across the life-course. The current study establishes that these barriers are pertinent for community-dwelling adults in Northern Ireland, and provides a rich understanding within this regional context. Several novel barriers to talking about death and dying were also identified, including a focus on interpersonal communication skills and cultural beliefs. The identification of facilitators to provide a more multi-dimensional understanding of the drivers of this behaviour was also a novel contribution of this study, with a previous lack of prior attention in the research literature [47].

The current study would suggest that societal norms place boundaries on the perceived opportunities for death talk, with respondents believing that these conversations should only take place within families and in particular circumstances. This is a significant constraint when individuals believe family members are not willing to engage in death talk, as is similarly reported by previous UK research [53]. There is indication of death talk as potentially a ‘limited taboo’ [54], with not ‘society’ per say but rather particular subgroups finding talk of death and dying challenging. It is unclear whether this relates to death as a psychological taboo, or rather suggests conversational embarrassment in engaging in death talk [55]. Indeed, a prominent theme in the current study describes respondent’s concern around the acceptability of emotional expression during these conversations with family and friends. This suggests that increasing awareness and accessibility of safe spaces such as Death Cafes for gentle discussion of death and dying with wider community members is valuable [56]. There is a dearth of formal evaluation on such initiatives [56], but conceptually the aim of Death Cafes includes supporting individuals to express emotion that may not feel able to do elsewhere, another key barrier reported in this study. There is suggestion that engagement with Death Cafes in the UK is currently dominated by middle-aged women working in healthcare [57], with a need to consider how such initiatives may be optimised to engage ‘hidden publics’ such as young people and men.

The perception that others are unwilling to engage in death talk relates to a key facilitator on the importance of normalising discussion of death and dying. Towards this goal, a life-course approach to discussing death and dying was suggested, and similarly proposed in terms of the need for education on the death system. Educational settings were cited as an opportunity to engage children and young adults, embedded within the context of life skills (i.e., equated to ‘sex education’ by respondents). Although there is a dearth of research on children’s perception of death, Paul [58] proposes a model of ‘death ambivalence’ where children are both death avoidant and death facing. The avoidance of death was however largely a result of the social domains the children were part of (family & education), in addition to wider cultural norms of what it means to be a child. There is an openness and desire for information and discussion of death from children [58], and recent research in Spain would indicate parents are favourable about inclusion of death education in their children’s schooling [59]. Recent research in Northern Ireland [60] also suggests value in integrating education on the death system in young adults’ university education, where a high level of awareness but lack of knowledge around palliative care is reported.

Respondents discussed concern about their interpersonal communication skills, which referred to both the respondent’s perception of their own skill and their perception of the skill of others to engage in meaningful conversations about death and dying. Although there was an identified need to encourage individual responsibility in initiating these conversations, this theme largely centred around equipping interested individuals with the ‘tools’ for engagement. There has been a focus on developing evidence-based peer-led ACP facilitator training programmes [61], involving either peers or lay volunteers. This has involved facilitating ACP conversations and advance care directive completion, and provision of ACP education, training, and support. The majority of this training is focused on enabling volunteers to facilitate ACP conversations with older adults or clinical populations [61], however there is an evidence-base on which to inform supportive programmes for individuals in the community to facilitate conversations with close persons. There are also existing public-facing initiatives in this area which could be highlighted as part of a larger programme of support, e.g., the ‘Conversation Starter Kit’ [62]. For future generations, a life-course approach to discussing death and dying in early education may negate the need for formal programmes if individual self-efficacy around having these important conversations is improved through exposure.

Respondents in this study discussed concern about death talk causing offence/distress to people with strong spiritual or religious beliefs, which hinders death talk because of perceived incongruity of perspectives. This finding may be particularly pertinent to Northern Ireland, a post-conflict society in which religion can form an important part of individual’s social identity which influences their attitudes towards ‘outgroup’ members and may have resulted in heightened sensitivity [63]. There is also however relevance to the UK population more broadly, with an increasingly multi-cultural society [64] and adults identifying as non-religious [65], resulting in communities which are increasingly diverse in relation to spiritual or religious beliefs. Increasing awareness of different belief systems was reported as a facilitator in the current study and would appear an important component in interpersonal communication skills training for contemporary society.

Despite the majority of UK adults reporting being comfortable discussing death and dying with family and friends [20], recent reports would indicate only a minority have engaged in a conversation about their end of life wishes with others [10]. Health behaviour change theory includes the COM-B model [33] which can help in identifying the sources of a behaviour, to inform behaviour change interventions. Adults report being comfortable discussing death and dying with family and friends [20] could refer to being willing to have these conversations (*COM-B; motivation*), confident in having these conversations (*COM-B; capability*) or able to have conversations in prescribed circumstances (*COM-B; opportunity*). The COM-B model recognises the complexity in behaviour change and proposes that motivation, opportunity, and capability all need to be present in order for an individual to engage in a behaviour. The current study identifies several barriers and facilitators to talking about death and dying, which map to the majority of the COM-B components, and furthermore the TDF [34]. This suggests that in attempting to encourage community-dwelling adults to change their behaviour towards engaging more in death talk, it is likely that multiple complex interventions are needed, supported by policy level directives. The sources of behaviour identified in the current study will be relevant to community-dwelling adults in Northern Ireland, but alignment with previous research indicates generalisability to the wider UK. As a research area in its infancy, it may be useful to consider how existing initiatives map on to the COM-B model to identify mechanisms of change which may influence outcome.

There are few ‘upstream’ interventions to encourage conversations about death and dying among community members in the general population. Abba and colleagues [66] in their systematic review identified 5 studies, with only one study [67] developed to directly encourage individuals to discuss death and dying with family and friends. The evidence-base in this area is limited in both size and quality [66], however there is indication that passive methods of providing information (e.g., public lectures) are unlikely to be as effective as participatory approaches. Indeed, the need for education is a facilitator cited in the current study, but only addresses one component (*COM-B; capability*) of the multi-component approach needed. There are various examples of more experiential initiatives taking place in practice, yet few are formally evaluated. There is however promising evidence from evaluation of such initiatives in recent years, which may be more likely to address multiple COM-B components. An example is *the Heart of Living and Dying* in Northern Ireland [68], a supported group conversation where community members are invited to reflect on what matters to them in living and dying to begin to plan ahead. These novel initiatives are reflective of the need for innovation in this area [56], with a variety of structural barriers to community empowerment [69]. The current study identifies a variety of stakeholders, services, and approaches to facilitate greater communication about death and dying which may inform further innovation. Beyond intervention approaches at community-level, behavioural economics may inspire population-level interventions which are more efficient and economical, and based on strategies already utlilised by UK public health governenments e.g. the Behavioural Insights Team (a UK-based global social purpose organization) [70]. Interventions rooted in behavioural economics can be applied to public health policy and population-level programmes, and typically focus on restructuring social and physical environments to *gently* endorse (or ‘nudge’) health-promoting behaviour [70]. General examples include reducing the cognitive burden of health information (e.g. simplication of information on advance care planning to reduce decision fatigue), making the default option favour the desired behaviour (death literacy a part of the school cirriculum), and priming the desired behaviour via a relevant and familiar source (e.g. opening up a conversation around death, dying or loss is modelled/ captured in an episode of a popular drama series). Interventions based on this approach target drivers of behaviour such as emotions and impulses, habits, and social norms *indirectly* [70]. Behavioural economics therefore presents a potentially powerful toolkit to influence decision-making around communication about death and dying by redesigning the choice architecture. Evidence surrounding the effectiveness of interventions based on behavioural ecominics in health contexts in general is lacking and thus we lack guidance on the appropriate design and evaluation of such interventions [71]. However, the identification of drivers of communication about death and dying, particularly those which are relevant to behavioural economics approaches (e.g. via indirect targeting of social norms, habits, and emotions), is a useful first step to informing the design of population-level behavioural ‘nudges’.

### Strengths and Limitations

This is one of a small number of studies to examine the drivers of why community-dwelling adults do not engage conversations around death and dying. This study represents a ground-up approach to identifying barriers to death talk, and uniquely identifies facilitators to present a more holistic understanding of the sources of behaviour. A novel application of health behaviour change theory is provided, which adds support to the growing utility of this approach in palliative and end of life care [e.g. 32;35–37]. This is the first step of systematically developing an evidence and theory-based intervention using the Behaviour Change Wheel [33]. The barriers and facilitators aligned to COM-B and TDF domains may be further mapped to intervention and policy functions, in using the BCW to systematically develop evidence and theory-based behaviour change interventions. The current study also has several limitations. A convenience sample was recruited via social media and is not representative of the population of the Northern Ireland, for example with an over-representation of respondents identifying as female and who have completed a higher education degree. Individuals without digital literacy skills would have been excluded. The sample does however include a largely non-clinical population, therefore addressing the need for more research with the full population of interest, including younger adults [14]. A survey design with two open-ended questions were used to enable recruitment of a large sample, however it is acknowledged that interviews or focus groups may have resulted in richer data. It also must be recognised that in developing behaviour change interventions, it is the recommended to specify the behaviour according to the AACTT framework; Action, Actor, Context, Target, and Time [72]. The behaviour in this study (*talking about death and dying*) was not specified in this level of detail, and so is reflective of broad drivers of the behaviour for the population across different contexts, similar to the application of behaviour change theory to implementation of ACT in a recent systematic review [37]. It is acknowledged that this study adopted a ‘wide-angle lens’ to exploring the topic at a population level, and so recommendations pertaining to population subgroups cannot be made, though are suggested in the narrative. A related limitation is that the focus of the study was on self-reported barriers and facilitators of death talk, without focus on individual differences which have been associated with not having discussed end-of-life wishes in previous research [10] such as male sex, young age, not being born in the UK or owning one’s own residence. This underlines the importance of value of research to identify modifiable risk factors in key subgroups of the population. The focus of the current study is one UK region (Northern Ireland), and so the findings are most relevant for tailoring interventions for this population. Although similarity with previous UK-based research would indicate generalisability, future research directly focused on identifying barriers and facilitators to talking about death and dying is needed to confirm if these drivers are relevant for the wider UK population.

## Conclusions

The current study identified barriers and facilitators to death talk in Northern Ireland, reflecting knowledge about death and dying, the integration of death talk into everyday life, interpersonal communication, and actors and systems with a role in supporting change. A consideration of why we are not having conversations around death and dying with those in our communities has never been as pertinent [73]. Not only is embedding a meaningful conversation around death in the community important for achieving a good death across different circumstances, but a greater awareness of death and physical distancing restrictions from the COVID-19 pandemic may have led to individuals reflecting more on their core values. Reflecting on values, preferences and goals is a core component of ACP [8], suggesting a timeliness for community-level public health interventions to encourage death talk among the public. Towards this goal, the findings from the current study provide a vital understanding of the key drivers of this public health behaviour. This novel understanding is ready to be applied by other researchers to systematically develop evidence and theory-based behaviour change interventions using the BCW, towards increasing individual engagement in death talk. It is possible that the COVID-19 pandemic will have influenced some of the identified barriers and facilitators, for example, for a subgroup of individuals there may now be more social opportunity for death talk. A follow-up study is currently underway to determine if and how this period of mass bereavement has impacted on community-led conversations about death and dying.

## Data Availability

The datasets used and/or analysed during the current study are available from the corresponding author on reasonable request.
